# Night Myopia Studied with an Adaptive Optics Visual Analyzer

**DOI:** 10.1371/journal.pone.0040239

**Published:** 2012-07-02

**Authors:** Pablo Artal, Christina Schwarz, Carmen Cánovas, Alejandro Mira-Agudelo

**Affiliations:** Laboratorio de Óptica, Universidad de Murcia, Murcia, Spain; Lund University, Sweden

## Abstract

**Purpose:**

Eyes with distant objects in focus in daylight are thought to become myopic in dim light. This phenomenon, often called “night myopia” has been studied extensively for several decades. However, despite its general acceptance, its magnitude and causes are still controversial. A series of experiments were performed to understand night myopia in greater detail.

**Methods:**

We used an adaptive optics instrument operating in invisible infrared light to elucidate the actual magnitude of night myopia and its main causes. The experimental setup allowed the manipulation of the eye's aberrations (and particularly spherical aberration) as well as the use of monochromatic and polychromatic stimuli. Eight subjects with normal vision monocularly determined their best focus position subjectively for a Maltese cross stimulus at different levels of luminance, from the baseline condition of 20 cd/m^2^ to the lowest luminance of 22×10^−6^ cd/m^2^. While subjects performed the focusing tasks, their eye's defocus and aberrations were continuously measured with the 1050-nm Hartmann-Shack sensor incorporated in the adaptive optics instrument. The experiment was repeated for a variety of controlled conditions incorporating specific aberrations of the eye and chromatic content of the stimuli.

**Results:**

We found large inter-subject variability and an average of −0.8 D myopic shift for low light conditions. The main cause responsible for night myopia was the accommodation shift occurring at low light levels. Other factors, traditionally suggested to explain night myopia, such as chromatic and spherical aberrations, have a much smaller effect in this mechanism.

**Conclusions:**

An adaptive optics visual analyzer was applied to study the phenomenon of night myopia. We found that the defocus shift occurring in dim light is mainly due to accommodation errors.

## Introduction

The human visual system has a remarkably high dynamic range, easily covering more than 10 log units in luminance. Although different mechanisms permit good quality of vision over such a large illumination range, there are also significant changes occurring to the eye in dim light. In particular, one phenomenon that has attracted interest over several centuries is that called night, or nocturnal, myopia [1], [2]. It is an increase of the power of the eye under conditions of reduced illumination, as compared with the situation in bright light. In practical terms, subjects become relatively more myopic in dim light. The enormous importance of this phenomenon resides in the many activities relying on human visual observations at night, from astronomy to surveillance. There was a time when the magnitude of, and procedures to correct, night myopia were considered military secrets. As early as 1789, Maskelyne reported the phenomenon and his attempts to correct it in his own eyes to improve astronomical observations [1]. Since that first report, night myopia has been re-discovered by different researchers. Lord Rayleigh [2], often acclaimed as the discoverer of night myopia, noted: *“I have found that in a nearly dark room, I am distinctly short-sighted. With concave spectacles of 36″ negative focus my vision is rendered much sharper, and is attended with increased binocular effect. On a dark night small stars are much more evident with the aid of the spectacles than without them”*. During the mid-twentieth century, and mostly during World War II, there were many studies devoted to quantify and better understand night myopia [3]–[5]. More recently [6], this topic had a renewed interest in the context of safety during night driving. However it was concluded that typical luminance conditions (not less than 1 cd/m^2^) were not dim enough to actually produce significant myopic shifts.

The magnitude of night myopia appears to be very variable among individuals and across different studies. Values ranging from negligible to as much as −4 D of myopic shift have been reported. Average values in most studies are around −1.5 D, a significant figure that would severely degrade the quality of the retinal image. Over more than a century, there was an open debate on the causes of night myopia because different experiments provided often conflicting results. From early on, spherical aberration was suggested as being mainly responsible [7]. The rationale was that under low luminance, the pupil dilates and the natural positive spherical aberration in the eyes [8], [9] would induce a myopic shift. Chromatic aberration was another proposed candidate to explain night myopia [10]. The typical values of longitudinal chromatic aberration in the eye [11], [12] in combination with the Purkinje effect could explain a measurable myopic shift, although most estimates suggested this effect could only play a partial role [4]. Other competing hypotheses included the error in accommodation occurring in dim light [13]. The eye would not have a relaxed accommodation for distant objects under low luminance, producing an apparent myopic shift which would explain night myopia [14]. A large number of experiments have been carried out to isolate these factors and to explain their actual contribution to night myopia. Unfortunately, in many cases the results were contradictory and none of the hypotheses clearly stands out as the key explanation. Other possible explanations proposed include the use of peripheral areas of the retina under low luminance that may have a distinct (and more myopic) refraction [15]. It has often been suggested that a combination of all the factors would actually produce the effect with different relative contributions for each subject.

Interestingly, after a history of more than a century on this topic, today most of the same doubts still exist. However, the development in the last years of advanced optical techniques that may be applied to the eye, notably wavefront sensing and adaptive optics, present an opportunity to elucidate what are the main causes for night myopia. Adaptive Optics (AO), a technique developed in astronomy to remove the effect of atmospheric turbulence from telescope images, has also been adapted to be used in the human eye [16]–[19]. One application was to obtain high resolution images of the retina, allowing the resolution of individual photoreceptors and other retinal cells in vivo [20]. Another important application of AO is to produce controlled optical aberration patterns in the eye, enabling new experiments to understand better the impact of the eye's optics on vision [21]. In particular, it is possible to address the intriguing question of what are the actual contributions of different factors in night myopia. We built a new experimental instrument, an adaptive optics visual analyzer operating in invisible infrared light, allowing subjects to view a stimulus under controlled conditions of luminance and other factors. The relative myopic shifts for different situations were measured to reveal the underlying causes of night myopia. The adaptive optics instrument actually permitted to perform the experiments on night myopia under experimental conditions that were never possible before. Subjects determined the best focus position for a variety of optical (modified aberrations) and luminance conditions.

## Methods

### Experimental setup

**Figure 1:**
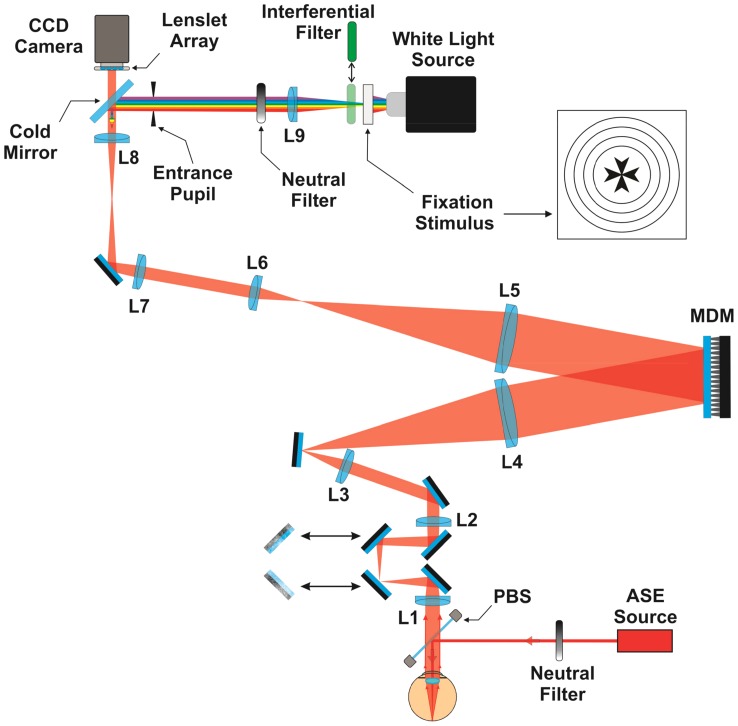
Schematic diagram of the experimental setup. See text for details.

A dedicated new instrument utilizing adaptive optics was built to determine subjects' best focus position under controlled optical conditions. A schematic diagram of the system is depicted in figure 1. It consists of a wavefront sensor to measure the eye's aberrations in real time and a correcting device, a deformable mirror, to modify the optics. A Hartmann-Shack (H-S) wavefront sensor [22] operating in invisible infrared light [23] measures the eye's aberrations and residual defocus (accommodation error) in real time (25 Hz). A narrow infrared beam (1050 nm; with a spectral spread of 50 nm) produced by an Amplified Spontaneous Emission source (ASE Broadband, BBS-1 µm. Multiwave Photonics, Portugal) is projected into the subject's retina acting as a beacon source. This wavelength is not visible allowing simultaneously measuring of the eye's optics while the subject performs visual tasks without disturbance. In the second pass, after the light is reflected in the retina and passes through the complete system, an array of lenslets (300 µm size and 6 mm focal length), optically conjugated with the subject's pupil plane, produces an image of spots on a CCD camera (C5999, Hamamatsu, Japan). The locations of the spots provide the local slopes of the ocular wavefront aberration. A 97-channel deformable mirror (DM97PMNRES4, Xinetics Inc., Devens MA, USA), with an aluminized glass faceplate and lead magnesium niobate (PMN) actuators, was used as the wavefront correcting device. It is placed in the system conjugated both with the subject's pupil plane and the wavefront sensor, by using appropriate sets of lenses in telescope configuration. Defocus in the system is controlled by moving two mirrors in a Badal optometer configuration. Subjects have access to the position of this optometer by means of a computer controlled micromotor stage. After lens L8, a cold mirror effects the transmission of the infrared light to the wavefront sensor while the visible light from a white light stimulus is directed to the eye. A green (550 nm with 10 nm spectral width) interference filter can be placed in front of the lamp to perform monochromatic light measurements. The AO system works in closed-loop at 25 Hz, with the deformable mirror driven by the measured wavefront aberration data. In the experiment, the deformable mirror was either passive, subjects operated with their normal aberrations, or was set to correct for each subject's spherical aberration. The system was operated first in closed-loop to reach the desired aberration values. Then the mirror kept that shape while subjects were performing the experiments. Subjects viewed a target stimulus (Maltese cross) printed on an overhead acetate and illuminated by a Xenon lamp (C7535/C4251, Hamamatsu, Japan). A set of neutral density filters was used to produce the desired luminance of the stimulus. The following conditions of luminance of the stimulus were selected: 1.35, −1.64, −3.14, −3.64, −4.14 and −4.64 Log(cd/m^2^). This range spans from photopic (around 20 cd/m^2^) to scotopic conditions (22×10^−6^ cd/m^2^). We measured the luminance of the stimulus plane and then the net values of luminance were estimated for each specific neutral density filter after considering the transmission of the system for the white light and monochromatic stimuli.

### Subjects and experimental conditions

Measurements were performed monocularly in eight subjects with normal vision. The range of age was 24 to 49 years old (average 33 years old SD = 7.5 years). Average refractive error was: mean sphere (−1.1 D, SD = 1.14 D) and cylinder (−0.2 D, SD = 0.27 D). Each subject was placed in a bite-bar looking at the stimulus. The eye's pupil was centered with respect to the apparatus by the operator by using an auxiliary camera (not shown in figure 1 for clarity). All the measurements were collected under normal viewing conditions, without cycloplegia. For each condition, subjects were asked to change the position of the Badal optometer to bring the stimulus to the optimum subjective visual focus. They started from a relative hyperopic position, but during each run, they could freely move the focus position in both directions. Five sequential repetitions for each condition were performed, taking the average and standard deviation. For each of the six luminance conditions, four comparative cases were tested: white light and monochromatic green light; and normal and corrected spherical aberration. During subjective assessment of the best focus, the eye's defocus (accommodation) was continuously recorded. This means that for each selection of best focus position, the actual accommodation lag or lead for the subject at that time was determined. Subjects operated with their natural pupil that varied with stimulus luminance and the accommodation error was estimated for a fix pupil diameter of 6 mm (calculating the equivalent in diopters from the value of the Zernike defocus term). The specific spherical aberration of each subject was corrected in one of the experiments. The average value of spherical aberration was 0.15 µm for 6 mm pupil. In the case of the low luminance conditions subjects were dark adapted for at least 30 minutes. The experiment room was maintained in complete darkness with the subject and one operator inside. The computers inside the laboratory running the experiment were remotely controlled by a computer in an adjacent room by using remote access control software (VNC, RealVNC Ltd., Cambridge, UK) through a LAN network. A second operator was in the adjacent room performing remote control of the whole process. Voice communication between the remote control room and the operator in the laboratory was achieved via Skype. This permitted the subject to maintain dark adaptation while the experiment was in progress. The use of the experimental setup and the complete procedure followed the tenets of the Declaration of Helsinki. Informed written consent was obtained by all subjects after they were fully informed about the nature and the possible consequence of the measurements. The study protocol was approved by the University of Murcia ethics committee.

**Figure 2:**
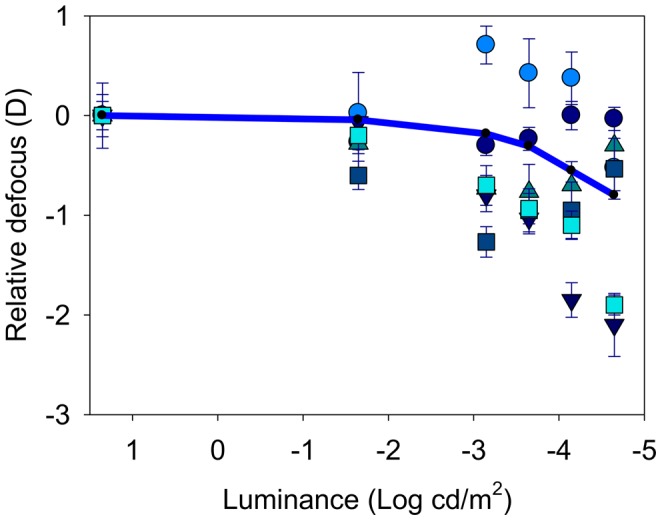
Relative defocus in diopters (D) as a function of the luminance of the stimulus (in Log(cd/m^2^). Individual symbols for each subject and luminance (error bars show 2 SD in the focus determination). The solid line in the figure is the average for all subjects.

**Figure 3:**
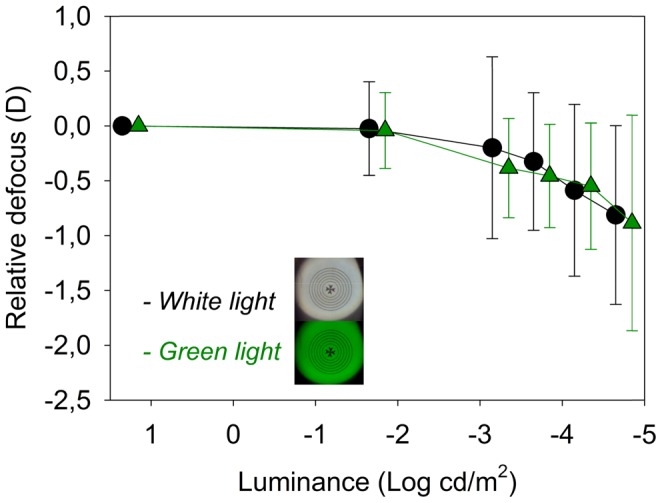
Average value of the relative defocus as a function of luminance in white light (black symbols) and in green monochromatic light (green symbols).

## Results

**Figure 4:**
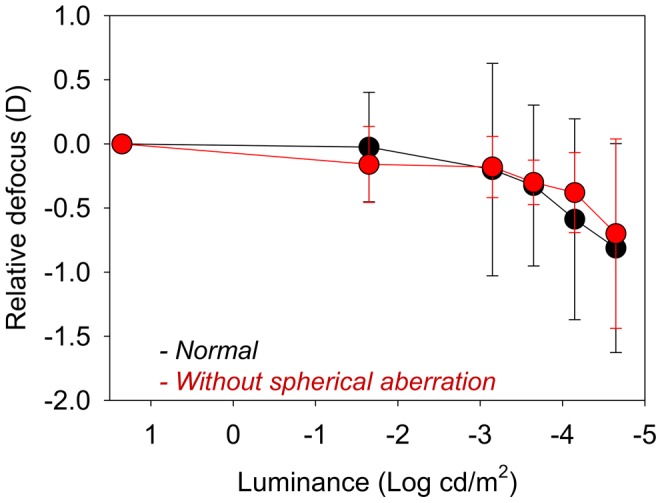
Average value of the relative defocus as a function of luminance with natural aberrations (black symbols) and with spherical aberration corrected (red symbols).

**Figure 5:**
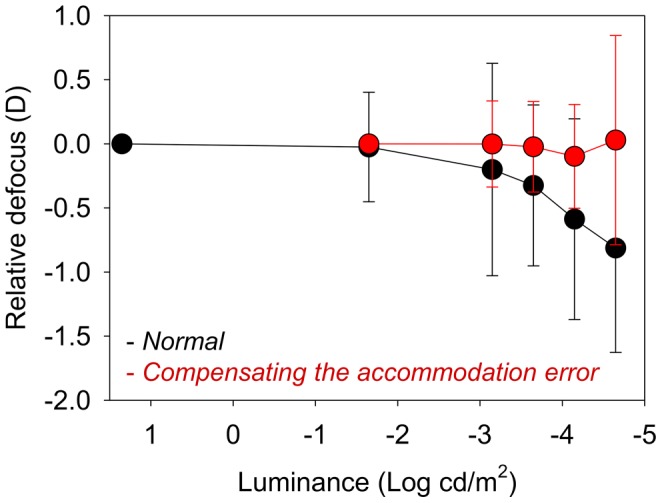
Average value of the relative defocus as a function of luminance when the accommodation error was considered (red symbols) as compared with the normal cases.

Figure 2 shows the results of defocus for each subject as a function of the luminance of the stimulus. In this graph, defocus is relative to the best focus at high luminance (20 cd/m^2^). First, data were collected in white light and natural aberrations. A large inter-subject variability is apparent from the results, with a range from 0 to −2.1 D in the measured defocus shift for the lowest luminance. The intra-subject variability determining the best focus in each condition ranged from 0.1 to 0.4 D, depending on the subject (error bars, representing standard deviations, are included for each individual focus determination). The solid line in the figure is the average for all subjects. For the lowest luminance level tested, the average myopic shift was −0.81 D (SD = 0.8 D). For other low light conditions, for instance 0.00022 cd/m^2^, a scotopic level, the average myopic shift was only −0.32 D (SD = 0.62) and around half of the subjects did not show a significant change of focus, with one even presenting a small hyperopic shift. For each subject, the same procedure for measuring the subjective best focus was repeated for the different experimental conditions to determine the underlying causes of the myopic shift phenomenon in dim light. Figures 3 and 4 show the average results in all subjects for the different experimental conditions. Figure 3 shows the average relative defocus in white light (black symbols) compared with the case in monochromatic green light (green symbols) for similar luminance levels of the stimulus. For both color lighting conditions, the results are undistinguishable indicating negligible impact of chromatic aberration. Figure 4 shows the average results comparing the defocus in white light wherein one case (black symbols) the normal aberrations are retained, and in the other (red symbols) the spherical aberration of each eye is corrected. Both defocus curves are similar which indicates that spherical aberration also only play a minor role in night myopia. The accommodation response was measured in real time using the Hartmann-Shack sensor in the setup when subjects were performing the focus setting experiment for each luminance. This allowed us to accurately determining any defocus shift due specifically to accommodation. The average defocus offsets for each subject and condition were estimated through a series of dynamic recordings. These objectively measured defocus values were compensated for each situation to evaluate the effect of accommodation lag in the myopic shift. Figure 5 compares the relative defocus with (red symbols) and without (black symbols) compensating the accommodation error. The average defocus shift is around zero when the accommodation error is incorporated.

## Discussion

We found that night myopia is a more elusive phenomenon than generally recognized. Despite the large body of evidence presents in the literature, our experiments performed under controlled conditions showed a large variability in our group of subjects and modest values of myopic shift at low luminance. In half of the subjects a myopic shift was not evident and the maximum shift was around −2 D in one subject with an average of −0.8 D. Inter-subject variability and dispersion of the results were common in previous studies. In some of them [24], a large number of subjects were tested providing up to 6 D range in myopic shifts in the dark. The conditions for that experiment were however very different. Based on our results, it seems that the practical importance of the phenomenon is more limited than was commonly believed. The small values reported in most subjects were only noticeable under very low luminance conditions, which are uncommon in ordinary conditions. In addition, dark adaptation was required for at least 20–30 minutes in complete darkness. For example, at luminance levels even lower than those occurring during night driving tasks (0.02 cd/m^2^), we did not find a defocus shift (−0.02 D, SD = 0.82 D). The inherent subjective nature of measuring refraction and the number of factors that may affect these determinations could provide an explanation to the variability and dispersion of the results in the studies of this problem. It should be noted that especially for the lowest luminance stimulus the task of finding the best focus was difficult for all subjects. However, the average standard deviation in the defocus estimates was 0.25 D in all subjects. It should be also mentioned that our measurements could be affected by some type of instrumental myopia. However, all the experiments were performed following the same procedure and within the same instrument. As we only compared differences, this should reduce most of the possible effect. In addition, the baseline subjective refraction results at high luminance were in good agreement with the purely objective measurements, not presenting any significant myopic bias.

Even recognizing the large variability within subjects, we decided to use the average relative defocus shift in all subjects as a metric to determine the contribution of different factors to night myopia. This was the main objective of this study and the experiments were planned to account for the three main proposed causes separately. The impact of chromatic aberration was evaluated by comparing the results obtained with white light (broad spectrum) and with monochromatic light. The average results showed no differences for all luminance levels. To better understand the expected impact of the chromatic aberration in our experiment, we calculated the theoretical shift by weighting the spectrum of the lamp used (see methods) with the photopic and scotopic sensitivity curves. The central effective wavelength was displaced by 43 nm to the blue region of the spectrum. In a simple eye model, this would be equivalent to around −0.2 D of myopic shift. Our average results did not even attain that predicted change. There is no doubt that the chromatic aberration of the eye's optics, combined with the wavelength dependent retinal sensitivity, may induce a small defocus shift at low luminance. However, this is a small value that could not explain night myopia alone.

Spherical aberration was initially proposed as responsible for night myopia. Although it is still mentioned in textbooks, the results were never solid. Our experiment was uniquely designed to test the impact of spherical aberration. We obtained the best focus positions at different luminance conditions with the normal spherical aberration in the subject's eye and when spherical aberration was corrected. The results were nearly identical showing that spherical aberration is not playing a significant role in night myopia. The rest of the aberrations present in the eye were not corrected in those experiments. An additional experiment where all the aberrations were corrected was also performed. Subjects reported an improved perception of the stimulus but the relative defocus as a function of luminance was similar.

The possible errors of accommodation in dim light have been suggested as a main possible cause for night myopia. Although results from experiments where accommodation was paralyzed, which should remove the effect, were conflicting, there were evidences in favor of this mechanism [14]. Our experiment and the specially developed optical apparatus provided for the first time the technical capabilities to completely determine at what extend accommodation errors played a role. It was possible to quantify the amount of defocus objectively measured as compared with the subject's subjective response. Although this part of the experiment also showed individual variability, the average relative defocus at low luminance conditions was completely accounted for by the errors in accommodation. This confirms this factor as the main responsible for night myopia. Anecdotally, it should be mentioned that the older (early presbyopic) subject participating in the study presented smaller values of both subjective myopic shift and accommodation. Our results also implicitly reduce the possible contribution of the other factors previously suggested. For example, changes in peripheral refraction at eccentricities of a few degrees should play only a minor role. This is in good agreement with recent high resolution refraction measurements in the periphery [25].

In summary, we performed a series of experiments allowing complete control of the optical conditions to measure the effect of luminance in the refractive state of the eye. This represents an interesting case-study in the use of state-of-the-art technology, an adaptive optics visual analyzer, to explain a classical phenomenon in vision, night myopia, that although extensively studied still lacked a complete understanding. We demonstrated that myopic shifts were modest and only occurred at very low light conditions and after dark adaptation. While clinically, defocus values as small as −0.50 D can produce visual symptoms, such refractive errors are exceeded in night myopia only under unusually low light conditions. This may imply a limited practical impact in most subjects although the situation under fully natural conditions, including binocularity would require future studies.
